# Induced cross-resistance of BRAF^V600E^ melanoma cells to standard chemotherapeutic dacarbazine after chronic PLX4032 treatment

**DOI:** 10.1038/s41598-018-37188-0

**Published:** 2019-01-10

**Authors:** Sarah Erdmann, Diana Seidel, Heinz-Georg Jahnke, Marie Eichler, Jan-Christoph Simon, Andrea A. Robitzki

**Affiliations:** 1 Centre for Biotechnology and Biomedicine (BBZ), Universität Leipzig, Division of Molecular Biological-Biochemical Processing Technology, Deutscher Platz 5, 04103 Leipzig, Germany; 2 Leipzig University Medical Centre, Department of Dermatology, Venerology and Allergology, Philipp-Rosenthal-Str. 23, 04103 Leipzig, Germany

## Abstract

The maximum response and 10-year survival rate for metastatic melanoma patients treated with standardised chemotherapy is still less than 15% and 10%, respectively. In contrast, oncogene targeting was found a promising tool for killing of BRAF^V600^ mutated melanoma cells. Nevertheless, despite improved response and survival rates, resistance acquisition remains an ongoing problem. In this context, the impact of chronic BRAF inhibition on the efficacy of commonly applied cytostatics is still unknown. In our study, human melanoma cells with BRAF^V600E^ mutation were treated with chemotherapeutics and a BRAF inhibitor. Resistance patterns were analysed by microelectrode array-based impedance spectroscopy, XTT and flow cytometric apoptosis/proliferation assay. BRAF^V600E^ melanoma cells acquired a time- and concentration-dependent desensitisation up to 100-fold towards oncogene-specific PLX4032 and chemotherapeutic dacarbazine after twelve months treatment. The impact of multiple drug insensitivity on molecular melanoma characteristics was elaborated via mRNA and protein quantification. Following BRAF^V600E^ targeting, melanoma cells developed an increasingly aggressive, dacarbazine-insensitive phenotype. Thereby, hyperactivated canonical alternative MAPK and bypass PI3K/AKT signalling caused cross-resistance of differently acting drugs. With these results, we are the first to show that long-term melanoma therapy with BRAF inhibitors can prevent further therapeutic success with dacarbazine due to acquisition of cross-resistance.

## Introduction

Due to intrinsic drug resistance or secondary desensitisation, therapy of patients with metastatic melanoma remains still a challenging task in cancer medicine^1^.

During the last decades, conventional mono- and polychemotherapy with anti-neoplastic drugs as dacarbazine or cisplatin was the common treatment for various cancer entities. Substantial limitations are response rates of only 5–12% and a low median overall survival of ten months, which is due to a rapid desensitisation by DNA damaging agents^2,3^. Interfered drug effects are caused by an increase in DNA repair, alterations in apoptosis and enhancement of survival/proliferation/invasion signalling (i.e. MAPK; PI3K/AKT)^4–6^.

A milestone in melanoma therapy was the clinical approval of vemurafenib (PLX4032) that specifically targets the mutated, constantly activated conformation BRAF^V600E^, a kinase in the mitogen-activated protein kinase (MAPK) pathway that is genetically modified in approximately 37–50% of all melanomas^7,8^. About 50–60% of patients initially responded to the mutation-specific therapy and showed improved median survival of 8–16 months^9^. However, again tumour cells established resistance mechanisms within six to eight months of chronic treatment^10^, which are mediated through the reactivation of survival signals. Activating mutations in protein kinase B (AKT)^11^ or phosphatidylinositol-4,5-bisphosphate 3-kinase (PI3K) as well as the loss of phosphatase and tensin homolog (PTEN) expression^12^ and the upregulation of receptor tyrosine kinases such as platelet-derived growth factor receptor β (PDGFRβ)^13^ promote PI3K/AKT signalling in a MAPK-independent manner. More common, MAPK-related mechanisms comprise BRAF^V600^ gene amplification^14^, development of neuroblastoma RAS viral oncogene homolog (NRAS) and/or mitogen-activated protein kinase kinase 1/2 (MEK1/2) mutations^15^ as well as mitogen-activated protein kinase kinase kinase 8 (COT) and CRAF overexpression^16,17^.

Until now, no study exists that examines the interrelation in resistance acquisition of currently applied oncogene targeting therapeutics and adjacent classical chemotherapy, which severely interferes with patients’ long-term survival. Here, we focus on the analysis of cross-resistance patterns in BRAF-mutated melanoma cells using microelectrode array-based impedance spectroscopy, a non-invasive, label-free bioelectronic method that was recently validated over standard XTT and ATP assays for the sensitive and comprehensive real-time detection of cellular drug effects *in vitro*^18–20^.

## Results

### Human melanoma cells harbouring BRAF^V600E^ mutation are sensitive to dacarbazine and PLX4032

**Figure 1:**
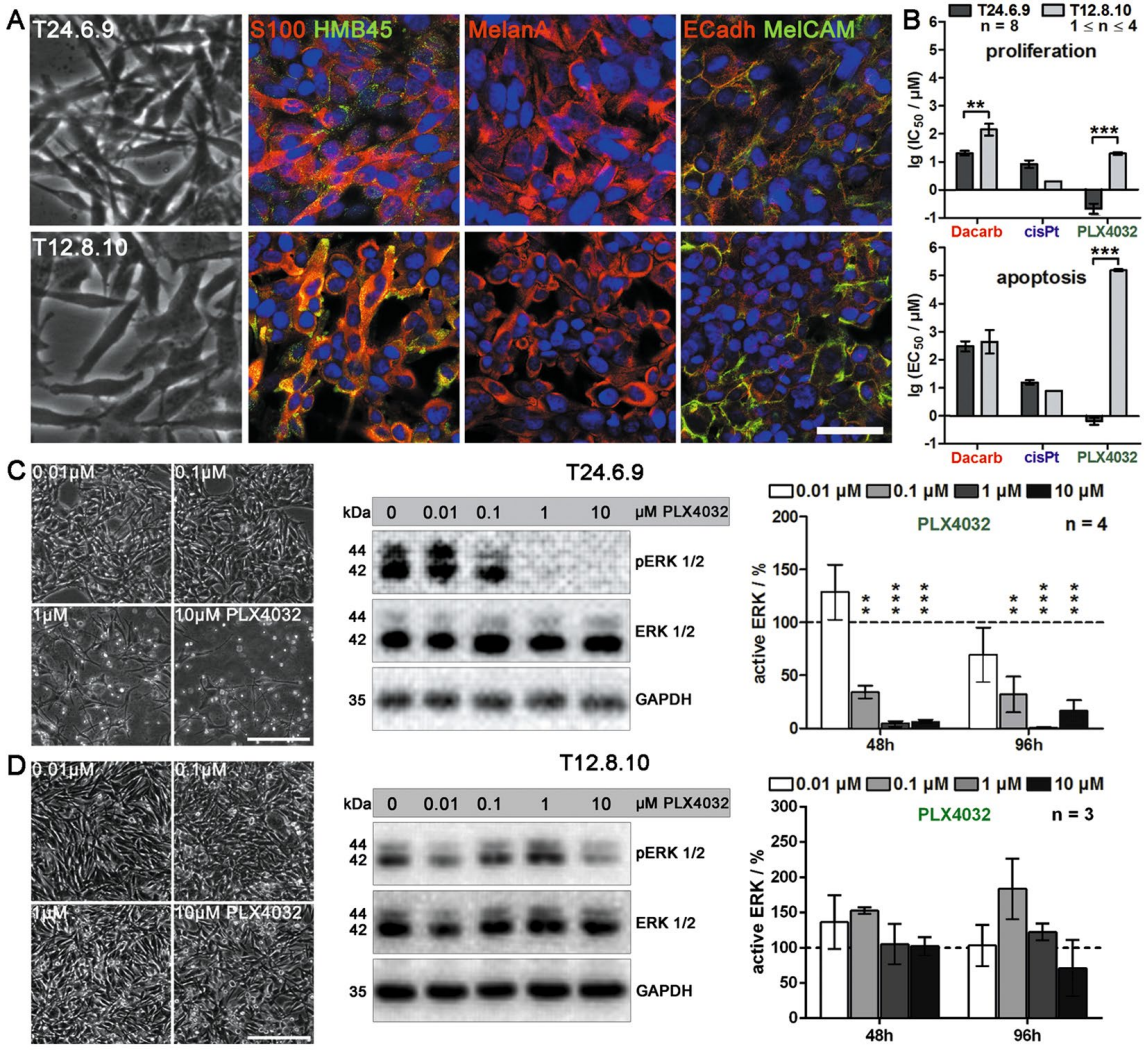
BRAF^V600E^ mutant T24.6.9 and wild type T12.8.10 melanoma cells are characterised by comparable melanoma characteristics but respond highly differently to BRAF inhibitor PLX4032 and chemotherapeutics dacarbazine and cisplatin. (**A**) Immunocytochemical staining of melanoma markers HMB45, MelanA and S100 as well as adhesion molecules E-Cadherin (E-Cadh) and MelCAM in human melanoma cells T24.6.9 and T12.8.10 (bar = 50 µm). (**B**) Flow cytometric analysis of proliferative and apoptotic cell populations (after 72 h treatment). Drug effects are displayed by logarithmised IC_50_ values. Effects of increasing concentrations of PLX4032 on T24.6.9 (**C**) and T12.8.10 (**D**) cells after 96 hours treatment (left: microscopic images (bars = 200 µm); middle: immunoblots). Quantification of active ERK (pERK) after 48 and 96 hours (right). Values are normalised to GAPDH expression and untreated control (dashed line, 100%). The blots were cropped to focus upon the specific proteins indicated. The entire gel blots are shown in Supplementary Figure S5. (n values depicted in figure; mean ± s.e.m.; **P < 0.01; ***P < 0.001); Dacarb = dacarbazine; cisPt = cisplatin; p = phosphorylated.

Human melanoma metastasis cells were treated with therapeutics under either acute (chemosensitivity) or chronic (chemoresistance) conditions. To assure relevance of the data for melanoma therapy, the drugs PLX4032, cisplatin and dacarbazine were added in concentrations that are clinically achievable^21–24^. Within this study, two melanoma cell lines, T24.6.9 and T12.8.10, were used. Tumorigenic origin was validated by melanocyte spindle morphology and expression of melanoma markers S100, HMB45 and MelanA as well as the adhesion molecules E-cadherin and MelCAM (Fig. 1A). DNA sequencing further revealed a BRAF^V600E^ mutation for the T24.6.9 melanoma population, whereas the T12.8.10 expressed the wild type gene.

Acute treatment of both cell lines with dacarbazine, cisplatin and PLX4032 led to substantial differences in proliferation and apoptosis indicating distinct efficacies of the tested drugs (Fig. 1B). The T24.6.9 showed considerably lower IC_50_ values (displayed as logarithmised values, lgIC_50_) and thus stronger impact of dacarbazine and PLX4032 on proliferation inhibition than T12.8.10, which in contrast, were more sensitive to cisplatin. Most substantially, application of PLX4032 induced IC_50_ values with a difference of almost 100-fold (Fig. 1B, upper panel). The apoptosis analysis confirmed these results, with EC_50_ values that showed a 10,000-fold difference, the effect of PLX4032 was even more convincing, (Fig. 1B, lower panel).

Morphologically, T24.6.9 cells showed apparent degeneration when exposed to 1 µM PLX4032 or higher (Fig. 1C, left panel). For those concentrations, immunoblotting revealed complete dephosphorylation of ERK 1/2 (Fig. 1C; middle panel). Quantitative analysis of active ERK 1/2 indicated a substantial decrease already induced by 0.1 µM PLX4032 for 48 hours (Fig. 1C, right panel), whereas total ERK 1/2 protein expression remained constant (Supplementary Figure S1). In contrast, the T12.8.10 cells showed no significant sensitivity towards PLX4032 up to 10 µM (Fig. 1D). Moreover, drug-dependent conformation change and RAF dimerization are known to cause the detected MAPK activation via ERK phosphorylation in BRAF^wt^ melanoma^25,26^.

**Figure 2:**
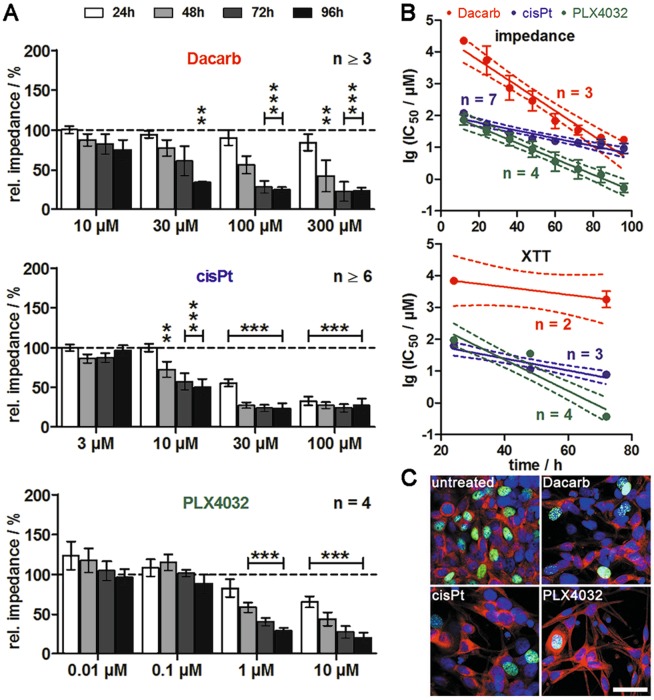
Cytotoxic potential of targeted therapeutic PLX4032 in BRAF^V600E^ melanoma cells T24.6.9 is considerably higher than for classical chemotherapeutics. (**A**) Impedimetric detection of concentration- and time-dependent chemosensitivity to dacarbazine, cisplatin and PLX4032. Values are normalised to experiment starting point and solvent control (dashed line, 100%). (**B**) Linear fitting and time-dependent linear progression of logarithmised IC_50_ values with 95% confidence intervals (dashed lines) assessed by impedance spectroscopy (upper panel) and XTT assay (lower panel). (**C**) Staining of 5-ethynyl-2′-deoxyuridine (EdU)-positive (green), proliferating cells in untreated and treated cultures (3000 µM Dacarb, 10 µM cisPt or 10 µM PLX4032) after 72 h (red: MelanA, blue: cell nuclei, bar = 50 µm). (n values depicted in figure; mean ± s.e.m.; **P < 0.01; ***P < 0.001); Dacarb = dacarbazine; cisPt = cisplatin; XTT = tetrazolium salt-based cytotoxicity assay.

For a time- and concentration-dependent analysis of drug effects on the BRAF^V600E^ mutant T24.6.9 melanoma cells, chemosensitivity was monitored by impedance spectroscopy^19,20^ via our self-developed planar interdigital electrode arrays^27^. First significant differences from control values could be observed for 30 µM dacarbazine after 96 hours (Fig. 2A, upper panel), whereas 10 µM cisplatin led to a significant impedance decrease already after 48 hours (Fig. 2A, middle panel). More strikingly, a similar progression could be observed for PLX4032 even at lower concentrations of 1 µM (Fig. 2A, lower panel).

To compare efficacy and kinetics of the applied substances, the impedimetric IC_50_ values were plotted over time and fitted linearly. The time-dependent linear decrease of the logarithmised IC_50_ values differed in slope and Y-interception, with PLX4032 leading to the overall lowest values indicating high efficiency, but a moderate degradation rate of −0.02 (Fig. 2B, upper panel). The T24.6.9 cells showed the lowest sensitivity to dacarbazine causing overall highest lgIC_50_ values, but with the fastest kinetics (slope: −0.04). In comparison, cisplatin activity was rather moderate. To validate impedance values, the cytotoxicity of the three compounds was assessed by standard XTT assay (Fig. 2B, lower panel). The values for PLX4032 and cisplatin lay within the range of the impedimetric results. However, the overall higher values and the reduced slope (−0.01) indicated less impact of dacarbazine treatment on metabolism and thus cytotoxicity. This finding was confirmed by nuclear proliferation staining showing stronger 5-ethynyl-2′-deoxyuridine (EdU) incorporation for dacarbazine compared to cisplatin and PLX4032 (Fig. 2C).

### Chronic administration of dacarbazine and PLX4032 leads to time- and concentration-dependent resistance acquisition

To investigate if chronic treatment leads to an acquisition of resistance, the BRAF^V600E^ mutant T24.6.9 cells were long-term treated with dacarbazine, cisplatin and PLX4032. The chronic administration of 10 µM dacarbazine (T24.6.9-10Da), 1 µM cisplatin (T24.6.9-1cP) or 0.1 µM PLX4032 (T24.6.9-0.1PLX) for twelve months led to an altered sensitivity pattern compared to parental cells (Supplementary Fig. S2). Whereas the parental cells showed a continuous decrease of relative impedance down to 30% after 96 hours incubation with 1000 µM dacarbazine, the T24.6.9-10Da exhibited only a decline down to 85% with substantial difference to parental cells. Application of 10 µM cisplatin to the parental T24.6.9 led to a major decrease of 50% compared to only 25% in chronically treated T24.6.9-1cP after 60 hours and thus slight desensitisation. The strongest effect could be detected by chronic treatment with 0.1 µM PLX4032. Whereas incubation of the parental cells with 10 µM led to a continuous decrease of relative impedance down to 20%, the T24.6.9-0.1PLX cells initially showed a drop of 15%, but then recovered to 95% indicating substantial drug resistance. Microscopic documentation validated the determined insensitivity patterns (Supplementary Fig. S2). All chronically treated cell lines were more confluent and less degenerated than the parental cells.

**Figure 3:**
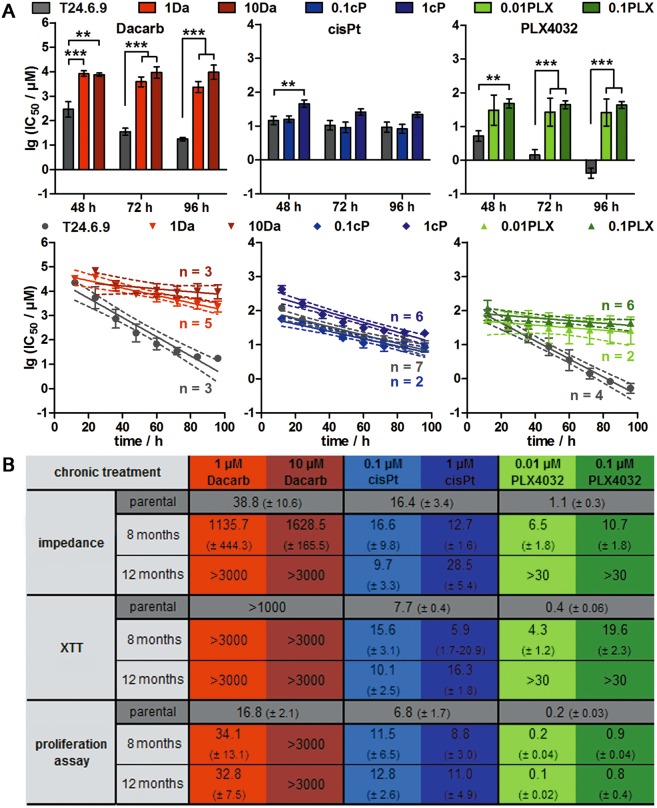
Chronic treatment of BRAF^V600E^ T24.6.9 melanoma cells with PLX4032 and dacarbazine induces a resistant phenotype with 100-fold increase of IC_50_ values in a time- and concentration-dependent manner. (**A**) Statistical analysis (upper panel) and linear fitting (with 95% confidence intervals [dashed lines]; lower panel) of impedimetrically determined IC_50_ values derived from parental and twelve months chronically treated T24.6.9 cells. (**B**) IC_50_ overview (µM after 72 h treatment) comparing parental and chronically treated cells concerning the time and concentration of treatment and the used method of data acquisition. (n values depicted in figure; mean ± s.e.m.; **P < 0.01; ***P < 0.001); Dacarb = dacarbazine; 1Da and 10Da = long-term 1 µM and 10 µM dacarbazine-treated cells; cisPt = cisplatin; 0.1cP and 1cP = long-term 0.1 µM and 1 µM cisplatin-treated cells; 0.01PLX and 0.1PLX = long-term 0.01 µM and 0.1 µM PLX4032-treated cells; XTT = tetrazolium salt-based cytotoxicity assay.

To figure out if desensitisation appears in a concentration- and time-dependent manner, parental and desensitised T24.6.9 (conditioned with two different drug concentrations for eight and twelve months) were treated with the respective drug. For chronic dacarbazine, a considerable increase of IC_50_ values by the 100-fold in a concentration-dependent manner could be observed (Fig. 3, upper panel). Long-term 1 µM, but not 0.1 µM, cisplatin treatment led to an IC_50_ value three times higher than for parental cells. As for dacarbazine, chronically PLX4032-treated T24.6.9 cells showed an overall significant increase of IC_50_ values. In contrast to cisplatin, the strong increase of IC_50_ values indicating resistance towards dacarbazine and PLX4032 was accompanied by 4–8 times lower degradation rates of the drug kinetics than in parental cells (Fig. 3A, lower panel).

Figure 3B gives an overview of all IC_50_ values obtained from different drug treatments and methods. T24.6.9 cells acquired a resistance to dacarbazine after twelve months chronic 10 µM application. No inhibitory effects on relative impedance, cell viability and proliferation were measurable any more. Chronic treatment with 1 µM cisplatin for twelve months only induced a negligible desensitisation characterised by twice the IC_50_ values of the parental cells. More strikingly, the acquisition of PLX4032 resistance in T24.6.9 after one year was comparable to the sensitivity of BRAF^wt^ T12.8.10 melanoma cells (Supplementary Fig. S3).

### Long-term treatment with dacarbazine and PLX4032 leads to cross-resistance

**Figure 4:**
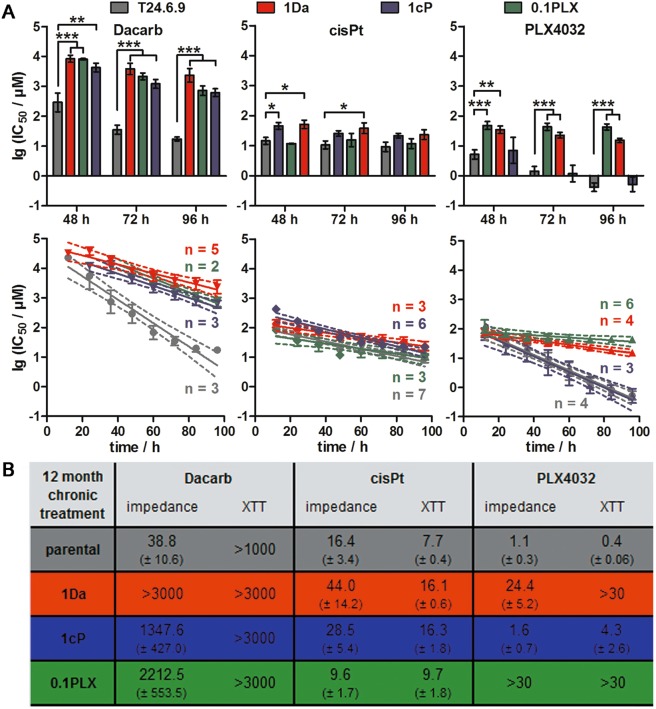
Chronically treated T24.6.9 melanoma cells develop a cross-resistance to both, dacarbazine and PLX4032. (**A**) Comparison of impedimetrically determined dacarbazine, cisplatin and PLX4032-induced IC_50_ values after acute treatment of parental and twelve months chronically treated (1Da, 1cP, 0.1PLX) T24.6.9 cells by statistical analysis (upper panel) and linear fitting of the logarithmised IC_50_ values (with 95% confidence intervals [dashed lines]; lower panel). **(B)** Overview of IC_50_ values (µM after 72 h treatment) determined by impedance spectroscopy and XTT assay comparing parental and twelve months chronically treated cells concerning their sensitivity towards dacarbazine, cisplatin and PLX4032. (n values depicted in figure; mean ± s.e.m.; *P < 0.05; **P < 0.01; ***P < 0.001); Dacarb = dacarbazine; 1Da = long-term 1 µM dacarbazine-treated cells; cisPt = cisplatin; 1cP = long-term 1 µM cisplatin-treated cells; 0.1PLX = long-term 0.1 µM PLX4032-treated cells; XTT = tetrazolium salt-based cytotoxicity assay.

To investigate if chronic treatment with dacarbazine, cisplatin or PLX4032 influences the efficacy of the other drugs, we analysed cross-sensitivity of the previously desensitised T24.6.9 cells. Therefore, the parental cells were compared with the chronically treated cells (1Da, 1cP, 0.1PLX) (Fig. 4A, upper panel). Whereas the native T24.6.9 was sensitive to dacarbazine, all three long-term treated cell lines were resistant indicated by 50–100 times higher IC_50_ values. Vice versa, dacarbazine resistance could be correlated with cross-desensitisation to cisplatin showing similar IC_50_ values compared to chronically cisplatin-treated cells. In contrast, no mutual resistance towards PLX4032 and cisplatin after long-term exposure was detected.

With increased IC_50_ values of up to 100-fold compared to parental cells, a clear resistance emergence of both 0.1PLX and 1Da cells to acute PLX4032 or dacarbazine application could be determined indicating cross-desensitisation. Similar to the resistant cells, cross-resistant cells showed not only higher IC_50_ values, but also smaller degradation rates of drug kinetics (Fig. 4A, lower panel). Similar effects detected by the XTT assay validated the observed cross-correlations as well as the applicability of different methods for chemosensitivity studies (Fig. 4B).

### Hyperactivated MAPK and PI3K/AKT signalling mediate cross-resistance between dacarbazine and PLX4032

**Figure 5:**
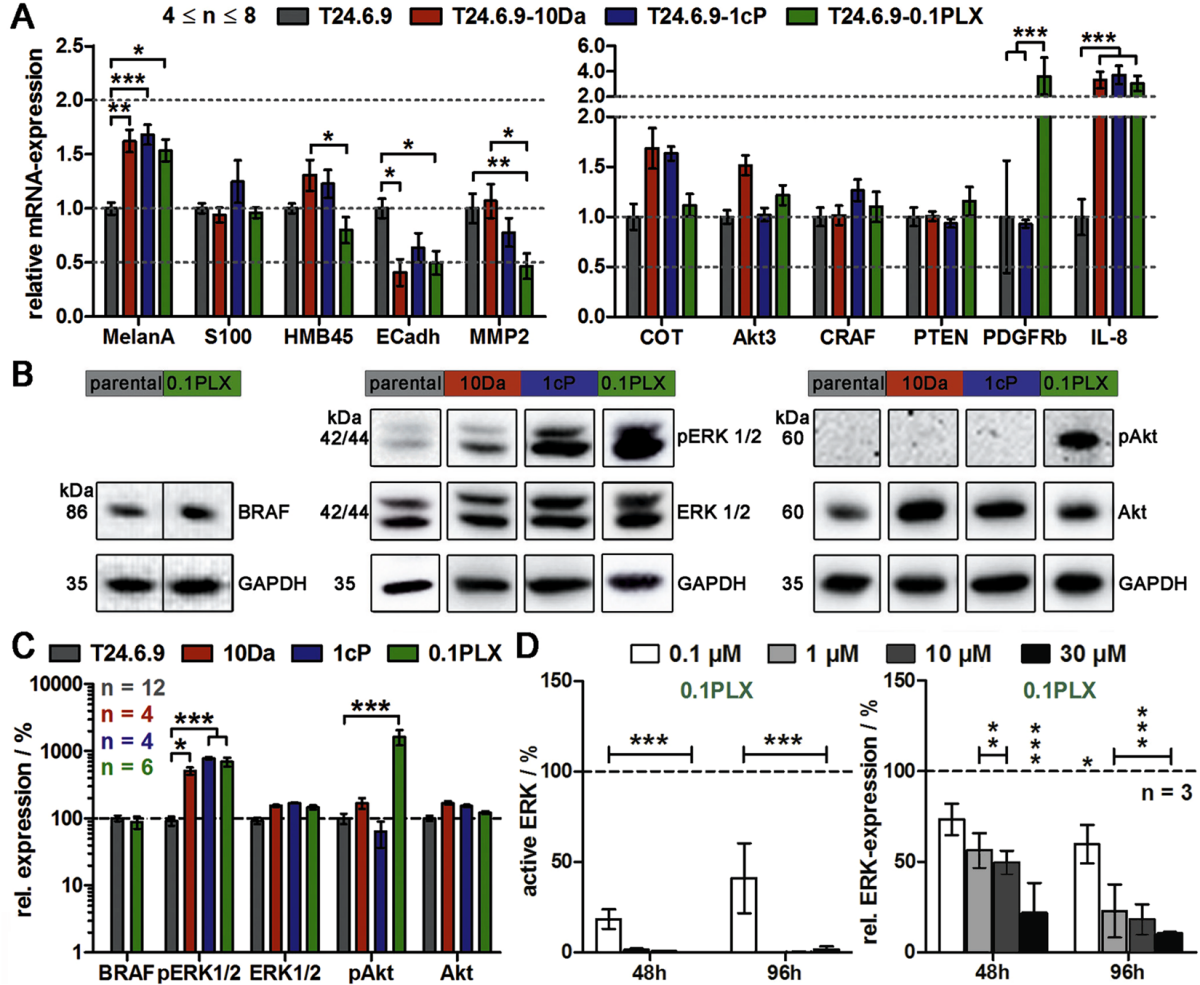
Long-term treatment with PLX4032 and dacarbazine induces cross-resistance via MAPK and PI3K/AKT/mTOR hyperactivation in more aggressive and invasive T24.6.9 melanoma cells. Parental cells (T24.6.9) in comparison to twelve months chronically treated cells (T24.6.9-10Da, T24.6.9-1cP and T24.6.9-0.1PLX) were analysed. (**A**) mRNA-expression levels of genes related to marker expression and cell migration (left) and chemoresistance (right). Values are normalised to housekeeping gene GAPDH and parental cells [=1.0]. Analysis of pathway signalling by protein (**B**) immunoblots and (**C**) quantification. Values are normalised to housekeeping gene GAPDH and parental cells [=100%]. The blots were cropped to focus upon the specific proteins indicated. The entire gels blots are shown in Supplementary Figs S6 and S7. (**D**) Relative activation (left) and expression (right) of ERK 1/2 during PLX4032 treatment of resistant 0.1PLX cells. Values are normalised to housekeeping gene GAPDH and the untreated, PLX-resistant control [=100%]. (n values depicted in figure; mean ± s.e.m.; *P < 0.05; **P < 0.01; ***P < 0.001) 10Da = long-term 10 µM dacarbazine-treated cells; 1cP = long-term 1 µM cisplatin-treated cells; 0.1PLX = long-term 0.1 µM PLX4032-treated cells; p = phosphorylated.

To examine possible mechanisms of cross-resistance, molecular expression and activation levels were assessed. The parental and chronically treated cells were analysed concerning the expression of genes related to melanoma marker expression, cell migration and chemoresistance (Fig. 5A). Predominantly, desensitisation led to increased expression (up to 1.5-fold) of melanoma markers (MelanA, HMB45). Furthermore, a substantial down-regulation of E-cadherin for all treatments and MMP2 down-regulation in 0.1PLX cells, both by 50%, could be detected. Elevated melanoma characteristics and decreased extracellular adhesion molecules indicated a more aggressive and invasive cancer phenotype in desensitised T24.6.9. Melanoma marker staining of parental and resistant 3D cultures further verified this assumption (Supplementary Fig. S4). Relative impedance of the resistant cell spheroids and their diameter was lower than for parental cells indicating less pronounced cell-cell contacts and smaller cell numbers, which are necessary alterations for invasiveness. Additionally, migration ability was tested and revealed a high migration potential especially of dacarbazine-resistant cell populations.

We then analysed genes within the MAPK and PI3K/AKT survival pathway. Most considerable differences were the clear elevation of IL-8 mRNA for all three desensitised populations and of PDGFRβ expression for PLX4032 resistant cells (3- to 4-fold changes). Subordinate effects were COT elevation (10Da, 1cP) and increase of AKT3 expression (10Da) (≈1.5-fold). CRAF and PTEN expression did not change.

To check for pathway activation, phosphorylation of the kinases BRAF, ERK1/2 and AKT were investigated by quantitative immunoblotting (Fig. 5B, C). An apparent change of ERK1/2 activation indicated by a substantial 5- to 8-fold increase of phosphorylation (total ERK1/2: 1.5-fold) for all three treatments was detected. In contrast, AKT hyperactivation by more than 10 times could only be observed in chronically PLX4032 treated cells (total AKT: 1.7-fold). Total BRAF expression was not affected. By showing that the PLX4032 concentration-dependent reduction of active ERK1/2 down to 0% in the 0.1PLX cells (Fig. 5D) did not induce cytotoxicity (Fig. 3), escape AKT/PI3K survival signalling was proven. Moreover, PLX4032 led to a concentration-dependent decrease of total ERK1/2 expression in the resistant but not parental cells (Supplementary Fig. S1). Furthermore, inhibitor studies of active ERK1/2 by SCH772984 and mTOR by AZD8055 were able to recover the sensitivity of 1Da and 0.1PLX cells towards PLX4032 and dacarbazine significantly. IC_50_ values decreased by more than 50% compared to the respective resistant phenotype proving that resistance signalling is realised via hyperactivation of MAPK and AKT/PI3K pathways (Supplementary Fig. S8).

## Discussion

The emergence of drug resistance is one main obstacle in today’s melanoma treatment. Due to high molecular adaptability, tumour cells can escape from apoptosis signalling within months^1^. The developed resistance often leads to a more aggressive tumour behaviour that, when it comes to cross-resistance, not only affects already applied drugs but also secondary therapies with dramatic impacts on the patient’s overall survival^28^.

**Figure 6:**
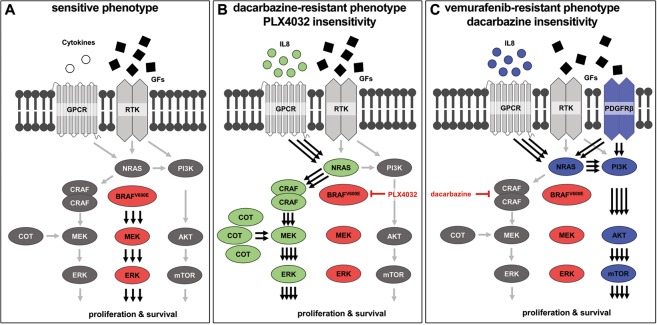
Dacarbazine and PLX4032 cross-resistance emerges via distinct MAPK-dependent and -independent pathway activation. Canonical and oncogenic MAPK and PI3K/AKT/mTOR signalling in (**A**) parental, sensitive BRAF^V600E^ melanoma cells, (**B**) dacarbazine-resistant BRAF^V600E^ melanoma cells that show cross-resistance to vemurafenib (PLX4032) and (**C**) PLX4032-resistant BRAF^V600E^ melanoma cells that have cross-resistance to dacarbazine. BRAF^V600E^ affected, hyperactivated kinases are marked in red, dacarbazine- and PLX4032-affected molecular targets of resistance emergence are marked in green and blue, respectively. GPCR = G-protein-coupled receptor, RTK = receptor tyrosine kinase, GFs = growth factors.

In this study, we compared two human melanoma cell lines that differed in their oncogene profile (BRAF^V600E^ and BRAF^wt^) causing altered sensitivities to classical chemotherapeutics and PLX4032, an inhibitor of mutated BRAF. Confirming the genetic modification, most substantial differences in the sensitivity of wild type and mutant populations were detected for acute PLX4032 treatment. In accordance with others^29^, IC_50_/EC_50_ values of proliferation/apoptosis assays varied by several 100-fold since PLX4032 only inhibited BRAF^V600E^-dependent MAPK hyperactivation (Fig. 6A). However, chronic drug treatment induced a desensitised BRAF^V600E^ melanoma phenotype within eight to twelve months dependent on the administered dose, which is similar to *in vivo* findings^30,31^. Moreover, we could achieve comparable or even higher levels of melanoma desensitisation than previous *in vitro* studies for PLX4032^32,33^ and dacarbazine^34^.

Excitingly, we are the first to describe the emergence of a more aggressive and invasive melanoma population, which is cross-resistant to PLX4032 and dacarbazine. Based on findings of drug resistance mechanism in recent studies, we provide results indicating that resistance of dacarbazine-conditioned BRAF^V600E^ cells to PLX4032 is mainly due to the re-activation of MAPK pathway by autocrine IL-8 cytokine stimulation^35–37^ of alternative MAPK signalling via COT and CRAF^16,17^ (Fig. 6B). In contrast, desensitisation of PLX4032-resistant melanoma to dacarbazine is caused by canonical MAPK-independent survival through IL8/PDGFRβ-dependent bypass signalling via AKT^38,39^ (Fig. 6C). The reversal of cross-resistance by selective inhibition of MAPK and AKT/PI3K pathway hyperactivation proved the proposed resistance mechanisms.

The paradoxical missing cross-resistance of cisplatin and PLX4032 depends on the mode of action of cisplatin in resistant and sensitive cells. Comparable to dacarbazine desensitization, cisplatin resistance might have emerged through ERK overactivation via COT signalling^40,41^. As this alternative pathway does not affect mutated BRAF, PLX4032 inhibition can still cause significant ERK deactivation and therewith, cell death. Contrary, elevated PI3K/AKT and MAPK signalling characterized PLX4032 resistance. Cisplatin is known to inhibit AKT phosphorylation during acute treatment^42,43^ and depends on ERK activation for apoptosis induction^44,45^, causing the cisplatin sensitivity of BRAF inhibitor resistant cells.

In this case study, the analysis of cross-resistance emergence between mechanically unrelated PLX4032 and dacarbazine, but not cisplatin was validated using one patient-derived BRAF^V600E^ melanoma cell line. Further investigation of the described co-desensitization events in other BRAF mutated cancer populations are needed to increase the relevance of the present findings for clinical purposes.

While commonly applied XTT or MTT *in vitro* cytotoxicity assays have a limited ability to detect cytostatic effects^46^, our non-invasive, label-free, real-time impedance technology allows precise and comprehensive chemosensitivity analysis in terms of both, cytotoxic and cytostatic compound screening^18–20^. Using impedance spectroscopy, we could identify time- and concentration-dependent cross-desensitisation patterns towards chemotherapeutic dacarbazine and BRAF^V600E^-targeting drug PLX4032 in BRAF-mutated metastatic melanoma cells that clearly correlated with the underlying activation/inhibition of MAPK- and PI3K/AKT survival signalling. Since treatment with chemotherapeutics is still the most common primary therapy in the majority of the world and generally indicated if the tumour shows insensitivity against targeted strategies such as BRAF inhibition^47^, our findings are particularly important and show the relevance of molecular melanoma characterisation prior to therapy in order to exclude cross-resistance effects and improve patient outcome^48^.

## Methods

A complete description of the methods is integrated in the Supplementary Material.

### Patient-derived melanoma cell lines

Viable patient-derived tumour tissue was obtained from excisions of cutaneous melanoma metastases. Therapeutically planned surgery was independent of the possible *ex vivo-*use of the clinically dispensable tumour specimens. All methods/procedures were performed in accordance to the Declaration of Helsinki with local ethics committee approval (Leipzig University Medical Centre; No. 224-11-11072011). In this context, written informed consent was always obtained from each patient before surgery. For detailed melanoma cell isolation, verification and maintenance, as well as resistance induction and reversal rational, consider Supplementary Methods.

### Chemotherapeutic agents

PLX4032 (S1267, Selleckchem); dacarbazine (LKT-D0011.1, Biomol); cis-diamineplatinum(II) dichloride (479306, Sigma-Aldrich); SCH772984 (S7101, Selleckchem); AZD8055 (S1555, Selleckchem).

### XTT assay

Metabolic activity was quantified using an XTT-based *In Vitro* Toxicology Assay Kit (Sigma-Aldrich). Full details are provided in the Supplementary Methods.

### DNA mutational analysis

BRAF mutation status was characterised using a primer pair of BRAF gene exon 15 (5′-ACTACACCTCAGATATATTTCTTC-3′; 3′-AATCAGTGGAAAAATAGCCTCAAT-5′). Full details are provided in the Supplementary Methods.

### mRNA expression analysis

Total RNA was isolated using the RNeasy Protect Mini Kit (Qiagen) according to the manufacturer’s protocol. RNA was reverse transcribed into cDNA applying the Accu Power CycleScript RT PreMix (dT20) (Bioneer) following distributor’s instruction. DNA was mixed with GoTaq® qPCR Master Mix (Promega) and the primers. The qRT-PCR was carried out in the Real-Time Thermal Cycler Rotor-Gene RG-3000 (Corbett Life Science). The applied primers are listed in Supplementary Table S1. For detailed information on qPCR cycling, quantification and quality control, see Supplementary Methods.

### Protein chemical analysis

For protein extraction, cell pellets were suspended in RIPA-buffer supplemented with Protease Inhibitor Cocktail (1:100, Sigma-Aldrich) and sonificated (Hielscher GmbH). The protein concentration was determined via a Bradford assay. After Western blotting and membrane blocking with 5% skimmed milk powder (Applichem), immunolabelling was realised using primary antibodies (all 1:1,000). Protein signals were detected applying horseradish peroxidase-conjugated secondary antibodies (all 1:5,000) and Nowa solution (MoBiTec), chemiluminescence was detected and quantified by the ChemiDoc-XRS documentation system (BioRad). The original gel blots are shown in Supplementary Figs S5–S7. Antibodies are listed in Supplementary Table S2. For detailed procedure, see Supplementary Methods.

### Flow cytometric analysis of proliferation/apoptosis

A TUNEL apoptosis assay using Terminal deoxynucleotidyl transferase (Promega, Germany) and Cy5-dUTP (GE-Healthcare, Germany) was used. For proliferation analysis, the Click-iT® EdU Alexa Fluor® 488 Flow Cytometry Assay Kit (Thermo Fisher Scientific) was applied according to the manufacturer’s protocol. Nuclei were stained with 15 nM POPO™-3 iodide (Life Technologies, Germany). Fluorescence of all samples was analysed with a BD FACS Calibur™ flow cytometer (BD Biosciences, Germany). Full details are provided in the Supplementary Methods.

### Immunocytochemistry

For immunocytochemical staining, fixed melanoma monolayer on coverslips and cryo-sliced spheroid sections were used. Cells were blocked with 3% BSA containing 0.1% Triton-PBS (all from Sigma-Aldrich). Afterwards, the sections were incubated with primary antibodies (anti-E-cadherin, 1:300; anti-Mel-5, 1:100; anti-melanosome, 1:300; anti-Melan-A, 1:500; anti-Mel-CAM, 1:100; anti-S100, 1:300) followed by incubation with fluorophore-coupled secondary antibodies (all 1:100). Finally, nuclei were stained with DAPI (1 μg/ml). Images were taken using a Nikon C1 plus confocal microscope (TE2000). Antibodies are listed in Supplementary Table S2. For detailed procedure, see Supplementary Methods.

### Impedance spectroscopy

Cells were seeded on 9-well interdigital electrode (9wIDE) arrays and measured at 50% optical confluence. Therefore, the 9wIDE MEAs were inserted in our self-developed multiplexer board connected to an Agilent 4294A high-precision impedance analyser (Agilent Technologies, USA). Impedance spectra (500 Hz to 5 MHz, 51 points, 10 mV amplitude) before and after drug application were recorded with our self-developed software IMAT v2.2.3. Additionally, blank values of cell-free 9wIDE MEAs were determined. Data analysis was done with the self-developed IDAT software v3.6, which calculates the relative impedance spectrum |Z|rel (%) = ((|Z|covered − |Z|blank)/|Z|blank × 100) and determines its maximum. Time traces of the relative impedance maximum were normalised to the starting point of the experiment to allow comparison and statistical analysis. Per experiment and condition, impedance of 5–7 electrodes was analysed. Full details are provided in the Supplementary Methods.

### Statistical analysis

All statistical analyses were performed using Graphpad Prism 5.02. IC_50_ values were determined by nonlinear sigmoidal curve fitting with normalised response and constant slope setting. For drug kinetics, logarithmised IC_50_ values were plotted over time and fitted linearly. Multiple group comparisons were done by a two-way ANOVA and Bonferroni *post hoc* test. All presented data is based on independent experiments. Normalisations and statistics are further defined in each figure legend.

## Supplementary information

Supplementary Information

## Data Availability

The datasets generated during the current study are available from the corresponding author on reasonable request.
