# Supplementary material for: Induced cross-resistance of BRAFV600E melanoma cells to standard chemotherapeutic dacarbazine after chronic PLX4032 treatment
Source: Sci Rep. 2019 Jan 10;9:30. doi: 10.1038/s41598-018-37188-0 (PMC6328535; doi:10.1038/s41598-018-37188-0)
Supplement: Supplementary file 1 — Supplementary Information [file 41598_2018_37188_MOESM1_ESM.docx]

**SUPPLEMENTARY INFORMATION**

**Induced cross-resistance of BRAF^V600E^ melanoma cells to standard chemotherapeutic dacarbazine after chronic PLX4032 treatment**

Sarah Erdmann, Diana Seidel, Heinz-Georg Jahnke, Marie Eichler, Jan-Christoph Simon and Andrea A. Robitzki

**SUPPLEMENTARY METHODS**

**Isolation of human** **patient-derived melanoma metastasis cell lines**

Sterile tumour specimens dissected from metastatic melanoma patients were placed into 10-ml vials containing RPMI basal medium with antibiotics and processed within four hours. Fat and surrounding connective tissue was extensively removed by scalpel. For isolation of tumour cells, the tumour tissue was minced using scalpels and seeded into T12.5 culture flasks in 1-mL complete growth medium (RPMI with 10 % fetal bovine serum, 1 % GlutaMAX supplement, 0.2 % Penicillin-Streptomycin, 0.04 % Gentamicin; all Fisher Scientific). After two to three days culture at 37 °C, 5 % CO_2_ and 95 % humidity, medium was exchanged and adhered cells were expanded for at least ten passages and mycoplasma-negative cell stocks were frozen for future use.

The line T24.6.9 was obtained at 24^th^ June 2009 from a melanoma metastasis biopsy, the line T12.8.10 was isolated at 12^th^ August 2010. Since cell isolation was done in our laboratories, initial cell authentication was not necessary. However, based on genome DNA mutation and mRNA analysis by human organism-specific primers, we could assure the human origin of these cell populations. Furthermore, frequent quality analysis of melanoma markers indicated the melanoma phenotype of the cells throughout experimental periods (see Figure 1).

**Resistance induction in patient-derived melanoma cell lines**

Resistance towards the chemotheroeutics dacarbazine and cisplatin as well as the BRAF inhibitor PLX4032 was induced by applying the drug concentrations also used in the acute treatment regime. Namely, those were for dacarbazine (0.1; 1; 10; 100 µM), cisplatin (0.1; 1; 10; 100 µM) and PLX4032 (0.01; 0.1; 1; 10 µM). The concentration ranges were derived from correlative studies that extrapolated the *in vivo/ex vitro* effect to the *in vitro* situation ^1-4^. The cells were then long-term cultured in the highest concentrations that the cells were able to tolerate (10 µM dacarbazine, 1 µM cisplatin and 0.1 µM PLX4032). The melanoma cells were treated for six to twelve months, and tested with regard to resistance and cross-resistance development after the specific incubation periods. The applied treatment periods correlated with or even extended the described time intervals for *in vivo* resistance emergence towards dacarbazine^5^, cisplatin^6^ and PLX4032^7^. The reversal of resistance was achieved by applying the ERK1/2 inhibitor SCH772984 at 100 nM with rising dacarbazine concentration (see above) and the mTOR inhibitor AZD8055 at 30 nM with rising PLX4032 concentration (see above).

**Gene mutation analysis**

Genomic DNA was isolated from cell pellets by Wizard® SV Genomic DNA Purification System (Promega). For Polymerase Chain Reaction (PCR), 9 µl of DNA was mixed with 10 µl GoTaq® qPCR Master Mix (Promega), and 0.5 µl of the primers (sense und antisense). PCR was carried out in the Real-Time Thermal Cycler Rotor-Gene RG-3000 (Corbett Life Science). PCR product identity (correct product size without unspecific products) was qualitatively controlled via agarose gel separation of qPCR products visualised by ethidium bromide. GeneRuler 100 bp Plus DNA ladder (cat.-nr. SM0322) was purchased from Thermo Scientific.

For sequencing, PCR products were mixed with 1 µl of sense primer as stated by service provider StarSEQ. Analysis of DNA mutations was carried out by alignment (MultAlin) of the theoretical DNA sequence of the gene (NC_000007.14; NCBI) with that identified by sequencing, with correlation to sequencing chromatogram.

**XTT assay**

Melanoma cells (40,000 cells/well) were seeded onto adherent 96-well plates. After incubation with compounds for 72 hours, the XTT solution was added for three hours at 37 °C, and the absorbance was measured at 450 nm with a Sunrise plate reader (Tecan). In addition, the absorbance at 690 nm was measured as a background and subtracted from the 450 nm value as well as the blank (in accordance to the manual).

**Quantitative PCR for mRNA expression analysis**

During qPCR, each of the 50 cycles comprised cDNA denaturation at 95 °C for 15 s, primer binding at 55 °C for 30 s, elongation at 60 °C for 30 s and fluorescence read-out at a product-specific temperature for 20 s. For quantification, the number of amplification cycles for the fluorescence SYBR® Green signal to reach a common threshold value (Ct) was analysed and fitted into a Ct standard curve of samples with known cDNA copy number. Per experiment and condition, duplicates were analysed.

All quantitative data as well as PCR product identity (correct product size without unspecific products) was qualitatively controlled via agarose gel separation of qPCR products visualised by ethidium bromide. GeneRuler 100 bp Plus DNA ladder (cat.-nr. SM0322) was purchased from Thermo Scientific.

**Protein chemical analysis**

For protein concentration analysis, Roti-Nanoquant (Carl Roth) was diluted 1:5 in *Aqua dest.* and transferred to the unknown samples. A bovine serum albumin-based calibration with known protein concentration was used for quantification. Optical density at 590/450 nm was measured with a microplate reader (Tecan) controlled by Magellan^TM^ (Magellan Software GmbH) and protein concentration was calculated. 25 µg protein was treated with Laemmli sample buffer, separated on a 10 % SDS polyacrylamide gel and blotted on a PVDF membrane applying 2 mA/cm^2^ for 50 min. Membranes were re-used several times for protein detection. Therefore, membranes were stripped and reloaded with antibodies against controls (non-phosphorylated kinases and housekeeping protein). Primary antibody incubation was carried out overnight at 4 °C.

**Flow cytometric analysis of proliferation and apoptosis**

Melanoma cells were harvested 72 h after drug application by treatment with 0.25 % trypsin/EDTA (Thermo Fisher Scientific) and 20 µg/ml DNase I Type II (AppliChem) for 5 min at 37 °C and mechanical dissociation. Subsequently, cells were washed with PBS (Thermo Fisher Scientific) and fixed with formaldehyde solution (4 % in PBS, 20 min; Carl Roth). After washing with PBS, samples were stored in 0.1 % triton/PBS at 4 °C (Triton X-100 from Merck).

DNA fragmentation as a marker for late apoptosis was studied by a TUNEL-Assay. Therefore, cells were permeabilised with 70 % ethanol for 30 min at -20 °C and washed. Incubation with 10 units TdT and 0.1 nmol Cy5-dUTP for 90 min at 37 °C was done to mark apoptotic cells.

**Immunocytochemistry**

For immunocytochemical staining, melanoma cells were seeded on collagen-coated (0.04 mg/ml in 0.02 N [acetic acid](http://www.chemspider.com/Chemical-Structure.171.html)) and phosphate buffered saline (PBS)-rinsed (both Thermo Fisher Scientific) glass coverslips and cultured to confluence. For fixation of adherent cells, 4 % formaldehyde solution (Carl Roth) was applied for 20 min, for spheroids fixation was prolonged to 1 h. After washing, monolayer cells were stored in at -20 °C, spheroids were stored in 20 % D(+)-saccharose solution (Carl Roth) at 4 °C. Spheroids were cryo-dissected using a LeicaCM 3050 S microtome with 20 µm slice width and transferred to microscope slides, dried and stored at -20 °C. After immunostaining, cells were dried and covered with Kaiser's glycerol gelatin (Merck).

**Spheroid generation**

T24.6.9 cells were seeded in non-adhesive 6-well plates at a concentration of 0.5 million/ml and placed on a horizontal gyratory shaker (72 rpm) in the cell culture incubator. After twelve days, the obtained 3D cultures were used for further experiments as immunocytochemical protein analysis, impedance spectroscopy and transwell migration assay.

**Cell migration assay**

For the analysis of chronic chemotherapeutic treatment on cell migration, transwell membranes with pore size of 8 µm were placed in 24-well plates. Wells were filled and membranes covered with complete growth medium. Parental and resistant monolayer cells were seeded on membranes at concentrations of 0.15 million cells/membrane. The number of migrated cells from the membrane to the bottom of the well was analysed after 144 h and cell nuclei staining with DAPI using a Nikon Eclipse TE 2000-U microscope with UV fluorescence filter. Per experiment and condition, triplicates were analysed.

**Fabrication of the 9-well-IDE microelectrode array**

Multiwell sensor-array in 96-well scale was produced in our clean room facility by standard lift-off technique. Therefore glass substrate surface (Borofloat 49/49/1.1 mm, Goettgens Industriearmaturen, Germany) was first cleaned in a cascade of ultrapure water, acetone, isopropanol and ultrapure water each for 2 min followed by a cascade of piranha etch/clean and ultrapure water removing organic remains. After drying glass substrate via spin-dryer and baking at a 95 °C hotplate for 5 min, spin-coating of positive resist (AR-P 3510, Allresist, Germany) was done as described in detail in the manufacturer's manual (Allresist, Germany), creating a coating thickness of 3 µm. After baking for 90 s at 95 °C, chip layout was passed on the substrate via photomask structure (Chromium mask, ML&C, Germany) using UV-light (380 nm, exposure time 13 s) (MA6 Mask Aligner, SÜSS MicroTec, Germany). A post-exposure bake step was performed before developing structure in positive resist AR-P 300-35 (Allresist, Germany), rinsing and drying substrate at 95 °C.

Direct current sputtering (CREAMET 500, CREAVAC GmbH, Germany) of 50 nm ITO (In_2_O_3_:SnO_2_, 90/10wt %, EVOCHEM, Germany) as adhesion layer followed by 350 nm gold as electrode material (BAL-TEC Preparation, Germany) was processed in an argon atmosphere at 4.0 × 10^−3^ mbar and 1.0 A/0.7 A. Positive resist and metal deposited thereon were removed afterwards in acetone, sensor-array was cleaned in isopropanol and ultrapure water prior to dehydration at 200 °C for at least 30 min.

To produce a cell adhesion-supporting surface as well as an insulation of the circuit paths, a passivation layer of negative tone photoresist NANO™ SU8‑2 (Micro Resist Technology, Germany) was used and processed via spin coating, pre-baking (1 min at 65 °C), softbaking (1 min at 95 °C), UV-light expose for 4.5 s, post-expose baking (1 min at 65 °C) softbaking (1 min at 95 °C) and development (1 min, MicroChem's mr-Dev 600, Allresist, Germany). The resultant film thickness of 1 µm of SU8-layer was verified by a confocal microscope (Micro Spy Topo, FRT GmbH, Germany). Finally, the microelectrode array (MEA) was cleaned in ultrapure water, spin-dried and plasma-cleaned at 400 mA for 7 min into the vacuum chamber (CREAMET 500, CREAVAC GmbH, Germany) at 2.5 x 10^‑2^ mbar with an Argon flow of 0.2 sccm. For paralleled conditioning, a 3 x 3 culture chamber in 96-well format (Greiner Bio-One, Germany) was bonded with polysiloxane onto the sensor array.

To ensure a consistent MEA quality, parameters as total number of functional electrodes, homogeneity of the passivation layer and cleanness of the electrode surface were controlled by a blank value impedance measurement in 0.1 % Triton in phosphate buffered saline (PBS).

**Impedance spectroscopy**

Prior to [cell](javascript:popupOBO('CL:0000000','C0LC00008F')) seeding, the wells of the sensor-arrays were coated by incubation with a [collagen](javascript:popupOBO('GO:0005581','C0LC00008F'))-solution (Thermo Fisher Scientific; 0.04 mg/ml in 0.02 N [acetic acid](http://www.chemspider.com/Chemical-Structure.171.html)) for at least one hour at 37 °C. After washing the wells with PBS (Thermo Fisher Scientific), 0.025 – 0.2 million melanoma cells were seeded in 200 µl complete growth medium per well on self-developed 9-well interdigital electrode (9wIDE) arrays and cultivated for three to four days until an optical cell layer confluence of 50 %.

Before starting the experiment, complete growth medium was renewed (300 µl/well) and the 9wIDE MEA was cultured another three hours. Prior to substance addition, impedance spectra were measured. Substance was added at the described concentrations per well and impedance was recorded once in an hour for 96 hours under constant culture conditions of 37 °C and 5 % CO_2_. For control [groups](javascript:popupOBO('CHEBI:24433','C0LC00008F','http://www.ebi.ac.uk/chebi/searchId.do?chebiId=24433')) the appropriate [solvent](javascript:popupOBO('CHEBI:46787','C0LC00008F','http://www.ebi.ac.uk/chebi/searchId.do?chebiId=46787')) was used. When measurement was finished, [cells](javascript:popupOBO('CL:0000000','C0LC00008F')) were removed from [electrodes](javascript:popupOBO('CMO:0002344','C0LC00008F')) by the use of 0.25 % trypsin/[EDTA](http://www.chemspider.com/Chemical-Structure.5826.html) solution (Thermo Fisher Scientific) and [spectra](javascript:popupOBO('CMO:0000800','C0LC00008F')) from [cell](javascript:popupOBO('CL:0000000','C0LC00008F'))-free [electrodes](javascript:popupOBO('CMO:0002344','C0LC00008F')) covered with 300 µl complete growth medium were recorded as blank values.

For spheroid cultures, our self-developed microcavity array (MCA)^8^ with pyramidal cavities (edge length 250 μm) in combination with the above mentioned impedance measurement platform was used. Impedance spectra (5 kHz to 5 MHz, 51 points, 100 mV amplitude) were recorded. After the initial measurement (0 h), the spheroids were individually transferred to 48-well plates containing the concentration of the tested chemotherapeutics and incubated under cell culture conditions on the horizontal gyratory (72 rpm) shaker for 72 hours. For discrete time points, spheroids were transferred manually to the measurement cavity of the MCA at room temperature, impedance spectra were recorded instantly (approximately five seconds per spheroid), and spheroids were transferred back to the gyratory shaker and cultured under cell culture conditions. Per experiment and condition, impedance of six spheroids was analysed.

**SUPPLEMENTARY REFERENCES**

1 Jahnke, H. G. *et al.* Direct chemosensitivity monitoring ex vivo on undissociated melanoma tumor tissue by impedance spectroscopy. *Cancer Res* **74**, 6408-6418, doi:10.1158/0008-5472.CAN-14-0813 (2014).

2 Ugurel, S. *et al.* In vitro drug sensitivity predicts response and survival after individualized sensitivity-directed chemotherapy in metastatic melanoma: a multicenter phase II trial of the Dermatologic Cooperative Oncology Group. *Clin Cancer Res* **12**, 5454-5463, doi:10.1158/1078-0432.CCR-05-2763 (2006).

3 Andreotti, P. E. *et al.* Chemosensitivity testing of human tumors using a microplate adenosine triphosphate luminescence assay: clinical correlation for cisplatin resistance of ovarian carcinoma. *Cancer Res* **55**, 5276-5282 (1995).

4 Yang, H. *et al.* RG7204 (PLX4032), a selective BRAFV600E inhibitor, displays potent antitumor activity in preclinical melanoma models. *Cancer Res* **70**, 5518-5527, doi:10.1158/0008-5472.CAN-10-0646 (2010).

5 Hauschild, A. *et al.* Dabrafenib in BRAF-mutated metastatic melanoma: a multicentre, open-label, phase 3 randomised controlled trial. *Lancet* **380**, 358-365, doi:10.1016/S0140-6736(12)60868-X (2012).

6 Burtness, B. *et al.* Phase III randomized trial of cisplatin plus placebo compared with cisplatin plus cetuximab in metastatic/recurrent head and neck cancer: an Eastern Cooperative Oncology Group study. *J Clin Oncol* **23**, 8646-8654, doi:10.1200/JCO.2005.02.4646 (2005).

7 Chapman, P. B. *et al.* Improved survival with vemurafenib in melanoma with BRAF V600E mutation. *N Engl J Med* **364**, 2507-2516, doi:10.1056/NEJMoa1103782 (2011).

8 Krinke, D. *et al.* A novel organotypic tauopathy model on a new microcavity chip for bioelectronic label-free and real time monitoring. *Biosens Bioelectron* **26**, 162-168, doi:10.1016/j.bios.2010.06.002 (2010).

**SUPPLEMENTARY TABLES**

**Supplementary Table S1: Applied human primers’ sequence and product length.**

| **Gene** | **Primer sense** | **Primer antisense** | **Product size** |
| --- | --- | --- | --- |
| AKT3 | AAGGGAAGAATGGACAGA | ATGGGTTGTAGAGGCATC | 136 bp |
| COT | CAAGTGAAGAGCCAGCAGTTT | GCAAGCAAATCCTCCACAGTTC | 154 bp |
| CRAF | GCACGGAGATGTTGCAGTAA | GCTACCAGCCTCTTCATTGC | 654 bp |
| E-Cadh | ATCCTCCGATCTTCAATCCCACCAC | GTACCACATTCGTCACTGCTACGTG | 270 bp |
| GAPDH | GACAGTCAGCCGCATCTTCT | AAATGAGCCCCAGCCTTCTC | 391 bp |
| HMB45 | CTGTGCCAGCCTGTGCTAC | CACCAATGGGACAAGAGCAG | 334 bp |
| IL8 | AGGGTTGCCAGATGCAATAC | AGCAGACTAGGGTTGCCAGA | 379 bp |
| MelanA | TCATCTATGGTTACCCCAAG | TCATAAGCAGGTGGAGCAT | 292 bp |
| MMP2 | GTGCTGAAGGACACACTAAAGAAGA | TTGCCATCCTTCTCAAAGTTGTAGG | 605 bp |
| PDGFRb | TTCCATGCCGAGTAACAGAC | CGTTGGTGATCATAGGGGAC | 91 bp |
| PTEN | GACAATCATGTTGCAGCAATTC | TTTGCCCCGATGTAATAAATATG | 87 bp |
| S100 | ACAAAGGAGGACCTGAGAGT | ACTGGTCCAGGTCCTTCATT | 106 bp |

**Supplementary Table S2: Applied human antibodies, supplier and catalogue number.**

| **Antibody** | **Supplier** | **Identifier** |
| --- | --- | --- |
| Rabbit polyclonal anti-Akt (phosphorylated Ser473) | Cell Signaling | 9271 |
| Rabbit polyclonal anti-Akt | Cell Signaling | 9272 |
| B-Raf | Cell Signaling | 9434 |
| Rabbit polyclonal anti-E-Cadherin | Cell Signaling | 3195 |
| Rabbit polyclonal anti-Erk1/2 (phosphorylated) | Cell Signaling | 9101 |
| Rabbit polyclonal anti-Erk1/2 | Cell Signaling | 9102 |
| Mouse monoclonal anti-melanosome (clone HMB-45) | Dako | M063429-2 |
| Mouse monoclonal anti-Mel-5 | Covance | SIG-38150 |
| Rabbit polyclonal anti-Melan-A | Abcam | ab51061 |
| Mouse monoclonal anti-Mel-CAM | Santa Cruz Biotech. | sc-80185 |
| Rabbit polyclonal anti-S100 | Dako | Z0311 |
| Rabbit polyclonal anti-GAPDH | Abcam | ab9485 |
| Goat polyclonal Alexa-488 IgG(H+L), anti-rabbit | Dianova | 111-546-144 |
| Goat polyclonal Cy3 IgG (H+L), anti-mouse | Dianova | 115-166-062 |
| Goat polyclonal HRPO IgG (H+L), anti-rabbit | Dianova | 111-035-144 |
| Goat polyclonal HRPO IgG (H+L), anti-mouse | Dianova | 111-035-003 |

**SUPPLEMENTARY FIGURES**


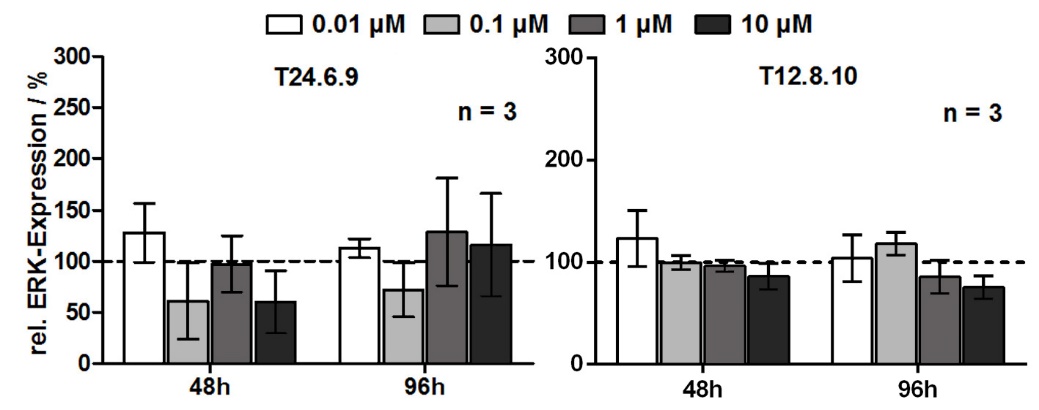


**Supplementary Figure S1: ERK1/2 expression of melanoma cells under PLX4032 treatment.** Quantification of relative Erk1/2 expression levels of T24.6.9 (left) and T12.8.10 (right) cells by immunoblotting. Values are normalised to GAPDH expression and untreated control (dashed line, 100 %). (n values depicted in figure; mean ± s.e.m.)

**
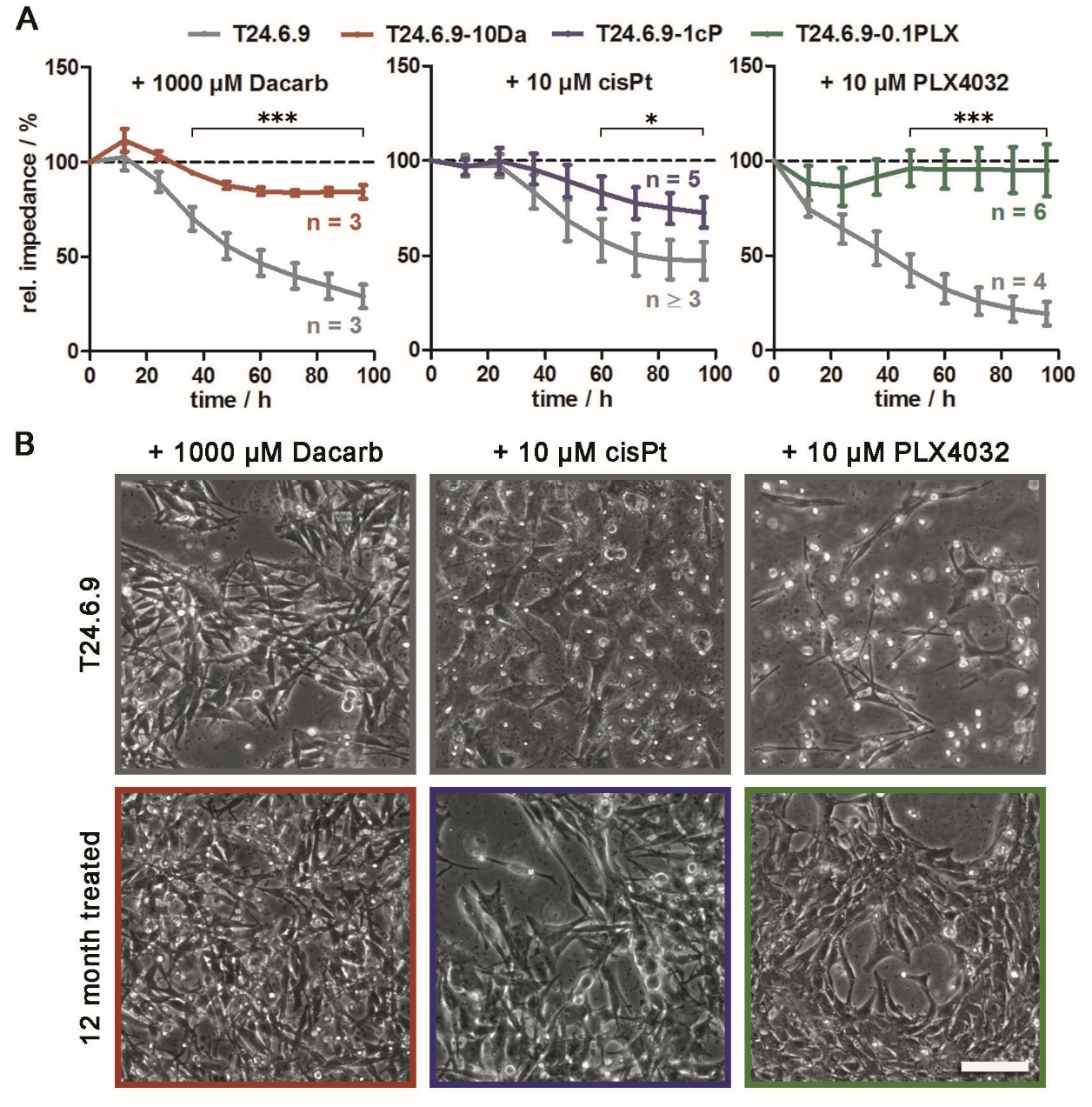
**

**Supplementary Figure S2: Resistance acquisition of chronically PLX4032-treated BRAF^V600E^ T24.6.9 melanoma cells is much more pronounced than for classical chemotherapeutics. (A)** Impedimetric chemosensitivity analysis of parental cells (T24.6.9) in comparison to long-term treated cells (T24.6.9-10Da, T24.6.9-1cP and T24.6.9-0.1PLX). Cells were monitored over 96 h after addition of either 1000 µM dacarbazine (Dacarb), 10 µM cisplatin (cisPt) or 10 µM PLX4032. Values are normalised to experiment starting point and solvent control (dashed line, 100 %). Significances indicate differences between parental and conditioned cells. **(B)** Cell morphology of parental and desensitised cells after 96 h treatment with either dacarbazine, cisplatin or PLX4032 (bar=100 µm). (n values depicted in figure; mean ± s.e.m.; *, P < 0.05; ***, P < 0.001); Dacarb = dacarbazine; cisPt = cisplatin.


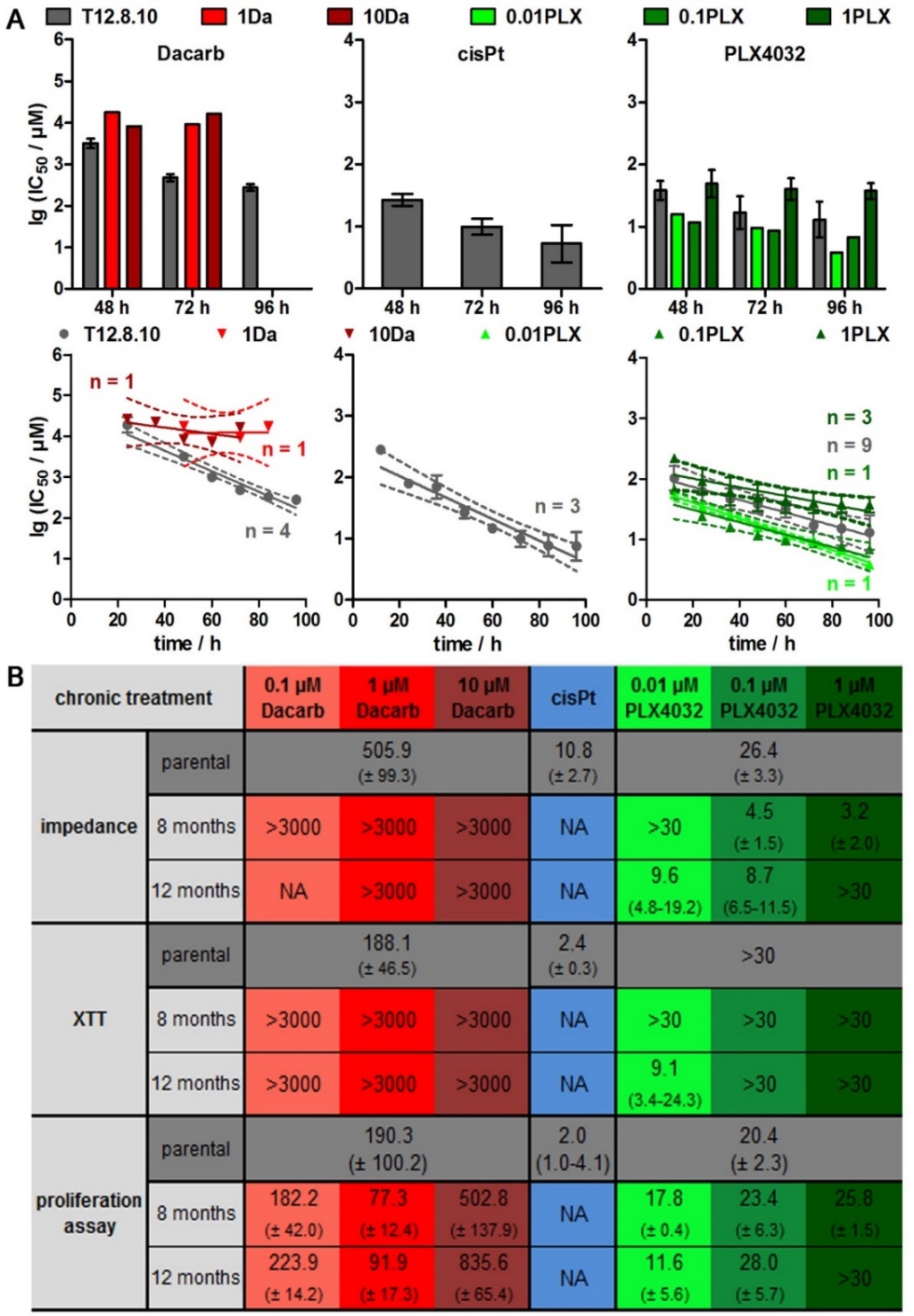


**Supplementary Figure S3: Time- and concentration-dependent desensitisation after long-term T12.8.10 treatment with dacarbazine, cisplatin and PLX4032. (A)** Statistical analysis (upper panel) and linear fitting (lower panel) of impedimetrically determined IC_50_ values with 95 % confidence intervals (dashed lines) derived from parental and eight months (with dacarbazine) and twelve months (with PLX4032) chronically treated T12.8.10 cells. For dacarbazine treatment, only for one of two experiments IC_50_ values could be determined because of low toxicity. After twelve months, no IC_50_values could be determined (n = 3). No long-term treatment with cisplatin could be performed, as the cells not even tolerated 0.01 µM. **(B)** IC_50_ overview (µM after 72 h treatment) comparing parental and chronically treated cells concerning the time and concentration of treatment and the used method of data acquisition. (n values depicted in figure; mean ± s.e.m.); Dacarb = dacarbazine; 1Da and 10Da = long-term 1 µM and 10 µM dacarbazine-treated cells; cisPt = cisplatin; 0.01, 0.1 and 1PLX = long-term 0.01 µM, 0.1 µM and 1 µM PLX4032-treated cells; XTT = tetrazolium salt-based cytotoxicity assay.

**
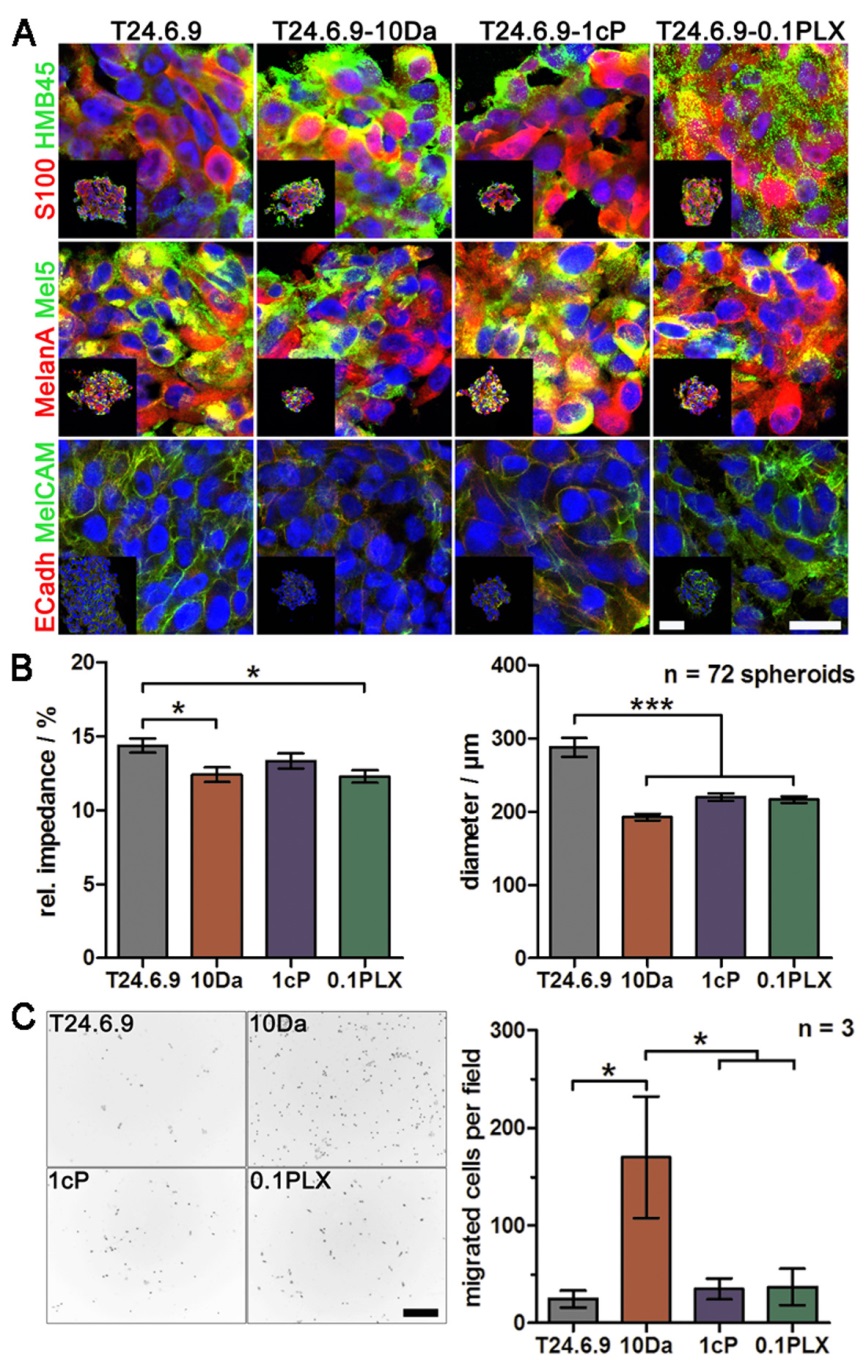
**

**Supplementary Figure S4: Impact of melanoma resistance on 3D culture properties and cell migration. (A)** Immunocytochemical staining of melanoma and invasiveness markers in spheroids derived from parental and twelve months long-term treated T24.6.9 cells (blue = cell nuclei). (small bar = 100 µm, large bar = 25 µm) **(B)** Impedimetric characterisation and diameter of melanoma 3D cultures performed with our self-developed microcavity array. **(C)** Qualitative and quantitative analysis of transwell migration of parental and chronically-treated monolayer cells after 144 h. Cell nuclei were stained using DAPI in the representative images (left). (n values depicted in figure; mean ± s.e.m.; *, P < 0.05; ***, P < 0.001); 10Da = long-term 10 µM dacarbazine-treated cells; 1cP = long-term 1 µM cisplatin-treated cells; 0.1PLX = long-term 0.1 µM PLX4032-treated cells.


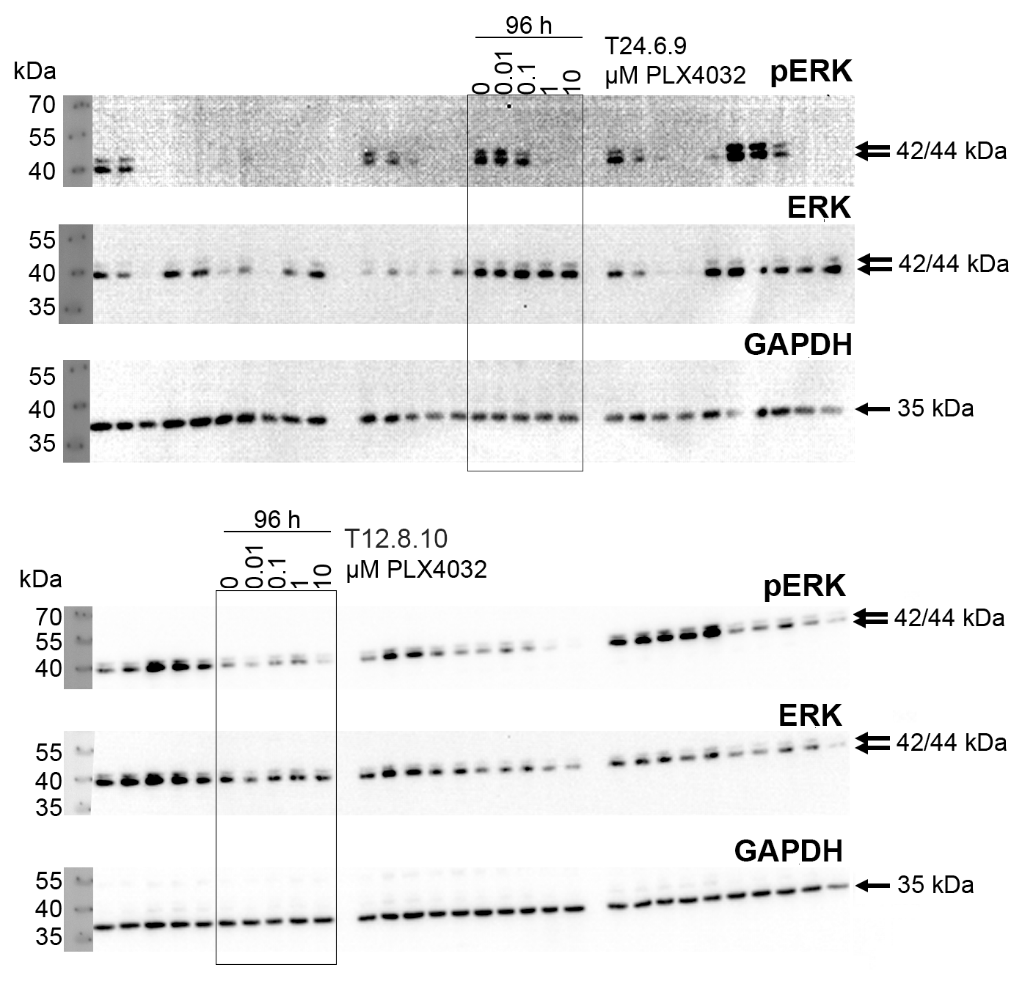


**Supplementary Figure S5: Original gel blots of PLX4032-treated T24.6.9 and T12.8.10 melanoma cells.** Activation of ERK was analysed. The framed parts were cropped and displayed in (top) Figure 1C and (bottom) Figure 1D.


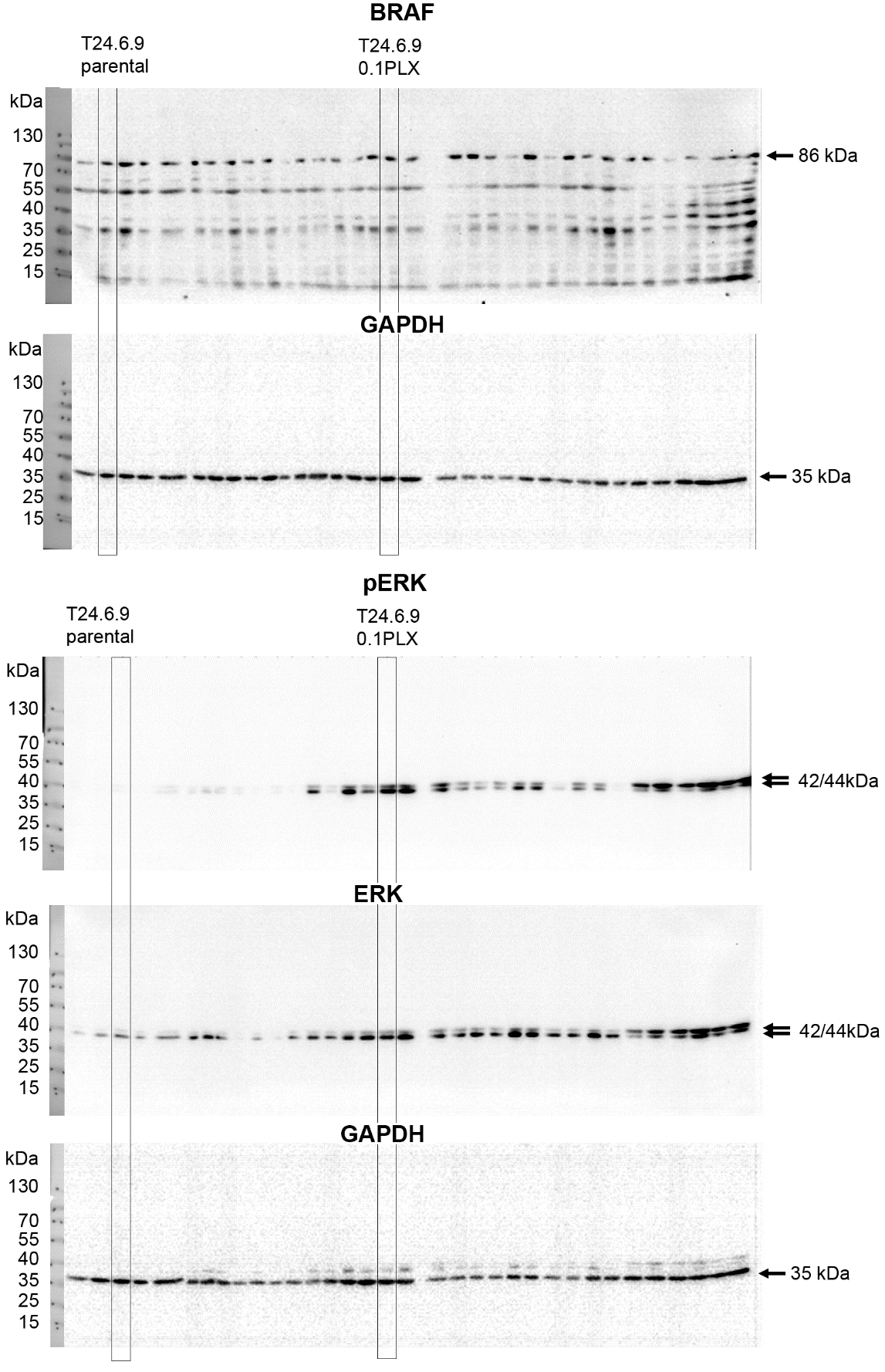


**Supplementary Figure S6: Original gel blots of native and long-term PLX4032-treated melanoma cells.** Resistance-dependent BRAF expression was analysed. The framed parts were cropped and displayed in Figure 5B. 0.1PLX = long-term 0.1 µM PLX4032-treated cells.


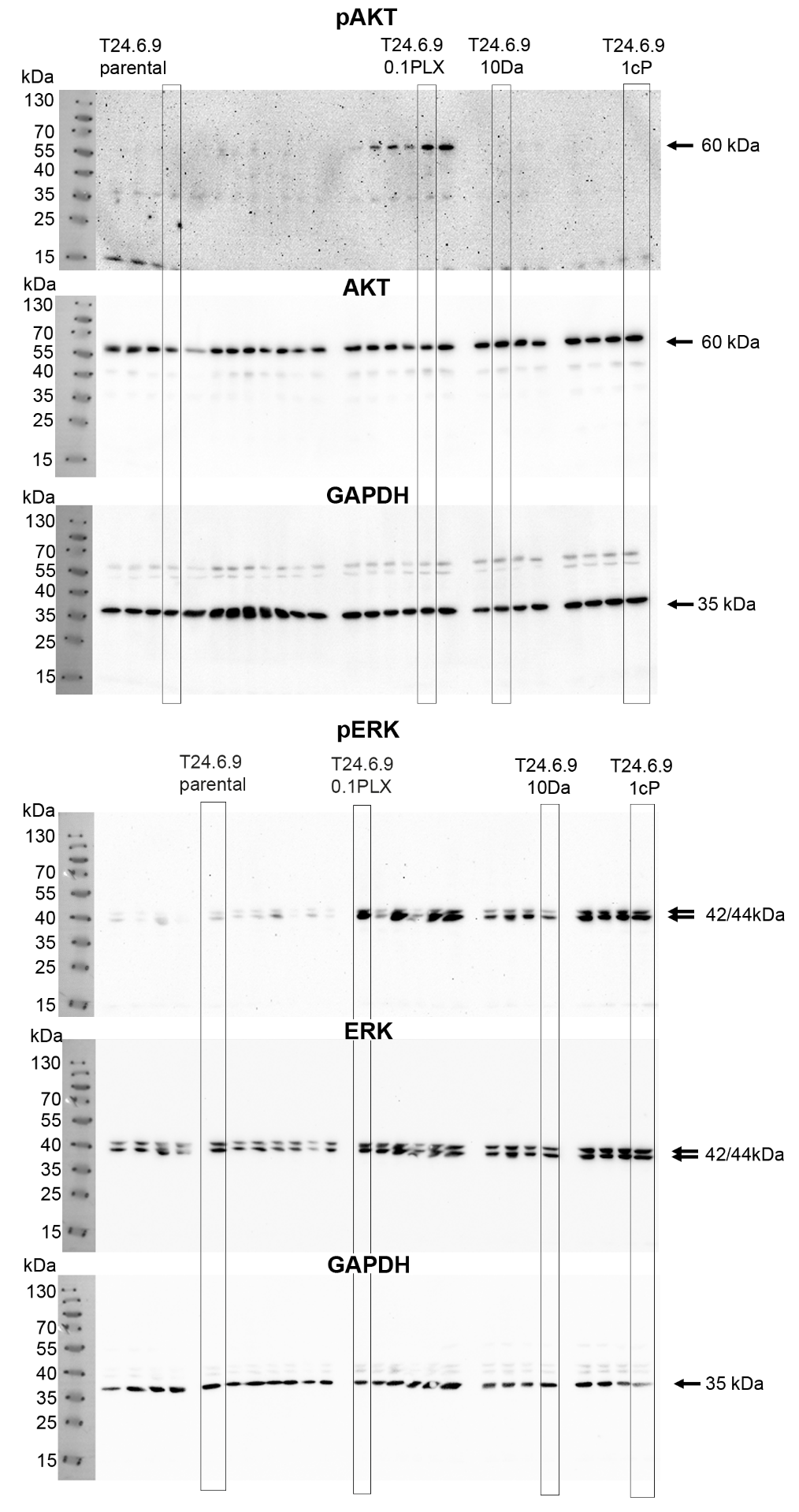


**Supplementary Figure S7: Original gel blots of native and long-term PLX4032-, dacarbazine- and cisplatin-treated T24.6.9 melanoma cells.** Resistance-dependent AKT and ERK activation was analysed. The framed parts were cropped and displayed in Figure 5B. 10Da = long-term 10 µM dacarbazine-treated cells; 1cP = long-term 1 µM cisplatin-treated cells; 0.1PLX = long-term 0.1 µM PLX4032-treated cells.


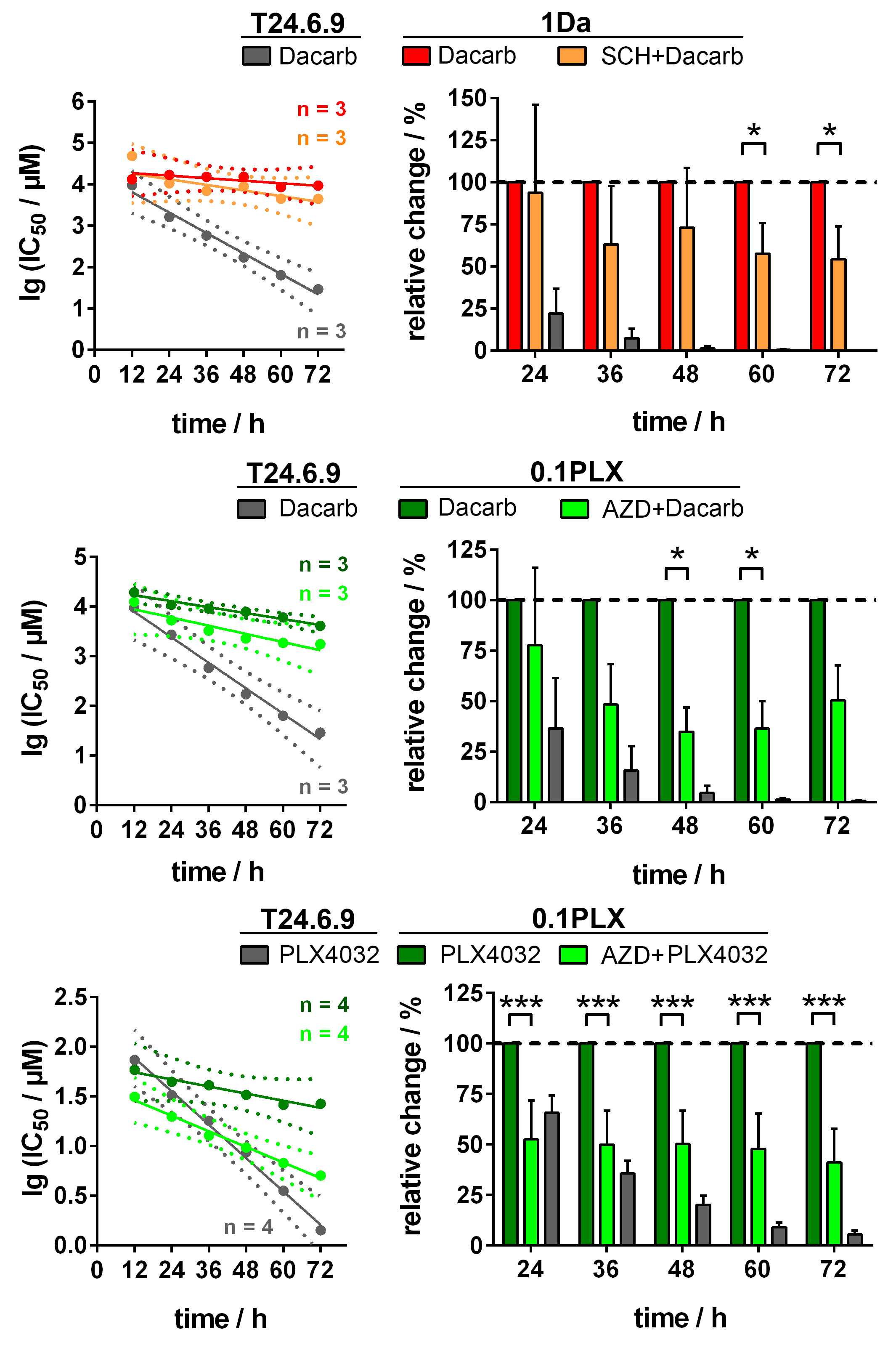


**Supplementary Figure S8: Selective inhibition of MAPK and AKT/PI3K hyperactivation in T24.6.9 cells cross-resistant towards dacarbazine and PLX4032.** Comparison of impedimetrically determined IC50 values of parental (grey) and twelve months chronically treated (1Da, 0.1PLX) T24.6.9 cells treated with PLX4032 or dacarbazine (Dacarb) alone or in combination with the ERK1/2 inhibitor SCH772984 (SCH) and the mTOR inhibitor AZD8055 (AZD). Linear fitting of the logarithmised IC50 values (with 95 % confidence intervals [dashed lines]; left panel) and relative change of the combined inhibition to the singularly PLX4032/dacarbazine-treated resistant phenotype and parental cells (right panel). (n values depicted in figure; mean ± s.e.m.; *, P < 0.05; **, P < 0.01; ***, P < 0.001); Dacarb = dacarbazine; 1Da = long-term 1 µM dacarbazine-treated cells; 0.1PLX = long-term 0.1 µM PLX4032-treated cells; AZD = AZD8055; SCH = SCH772984.
